# Polyphenols as Immunomodulatory Compounds in the Tumor Microenvironment: Friends or Foes?

**DOI:** 10.3390/ijms20071714

**Published:** 2019-04-06

**Authors:** Chiara Focaccetti, Valerio Izzi, Monica Benvenuto, Sara Fazi, Sara Ciuffa, Maria Gabriella Giganti, Vito Potenza, Vittorio Manzari, Andrea Modesti, Roberto Bei

**Affiliations:** 1 Department of Clinical Sciences and Translational Medicine, University of Rome “Tor Vergata”, 00133 Rome, Italy; chiara_focaccetti@hotmail.com (C.F.); monicab4@hotmail.it (M.B.); saramhh@hotmail.it (S.C.); giganti@med.uniroma2.it (M.G.G.); vitopotenza2@virgilio.it (V.P.); manzari@med.uniroma2.it (V.M.); modesti@med.uniroma2.it (A.M.); 2 Oulu Center for Cell-Matrix Research, University of Oulu, FIN-90014 Oulu, Finland; valerio.izzi@oulu.fi; 3 Department of Experimental Medicine, University of Rome “Sapienza”, 00164 Rome, Italy; sarafazi@hotmail.it

**Keywords:** polyphenols, cancer, immune response, inflammation

## Abstract

Polyphenols are natural antioxidant compounds ubiquitously found in plants and, thus, ever present in human nutrition (tea, wine, chocolate, fruits and vegetables are typical examples of polyphenol-rich foods). Widespread evidence indicate that polyphenols exert strong antioxidant, anti-inflammatory, anti-microbial and anti-cancer activities, and thus, they are generally regarded to as all-purpose beneficial nutraceuticals or supplements whose use can only have a positive influence on the body. A closer look to the large body of results of years of investigations, however, present a more complex scenario where polyphenols exert different and, sometimes, paradoxical effects depending on dose, target system and cell type and the biological status of the target cell. Particularly, the immunomodulatory potential of polyphenols presents two opposite faces to researchers trying to evaluate their usability in future cancer therapies: on one hand, these compounds could be beneficial suppressors of peri-tumoral inflammation that fuels cancer growth. On the other hand, they might suppress immunotherapeutic approaches and give rise to immunosuppressive cell clones that, in turn, would aid tumor growth and dissemination. In this review, we summarize knowledge of the immunomodulatory effects of polyphenols with a particular focus on cancer microenvironment and immunotherapy, highlighting conceptual pitfalls and delicate cell-specific effects in order to aid the design of future therapies involving polyphenols as chemoadjuvants.

## 1. Introduction

Polyphenols are a considerable group of natural compounds ubiquitously expressed in plants. They represent secondary metabolites with a major role in the growth, signaling, host defense against pathogens and ultraviolet [1]. They are found in foods and beverages of plant origin including fruits, vegetables, spices, cereals, nuts, legumes, olives, tea, coffee, and wine [2]. Different studies have shown that polyphenols have anti-inflammatory, anti-microbial, anti-tumoral and immunomodulatory properties, and therefore they can bring important benefits to human health [3]. Indeed, several investigations demonstrated the ability of polyphenols in regulating the human immune system, by affecting the regulation of the immune cells, the production of cytokines and other factors of the immune defense system [4].

Thus, the aim of this paper is to review the current knowledge of the immunomodulatory effects of polyphenols with a particular focus on cancer microenvironment and immunotherapy, highlighting conceptual pitfalls and delicate cell-specific effects in order to aid the design of future therapies involving polyphenols as chemoadjuvants.

## 2. Classification of Polyphenols

**Figure 1:**
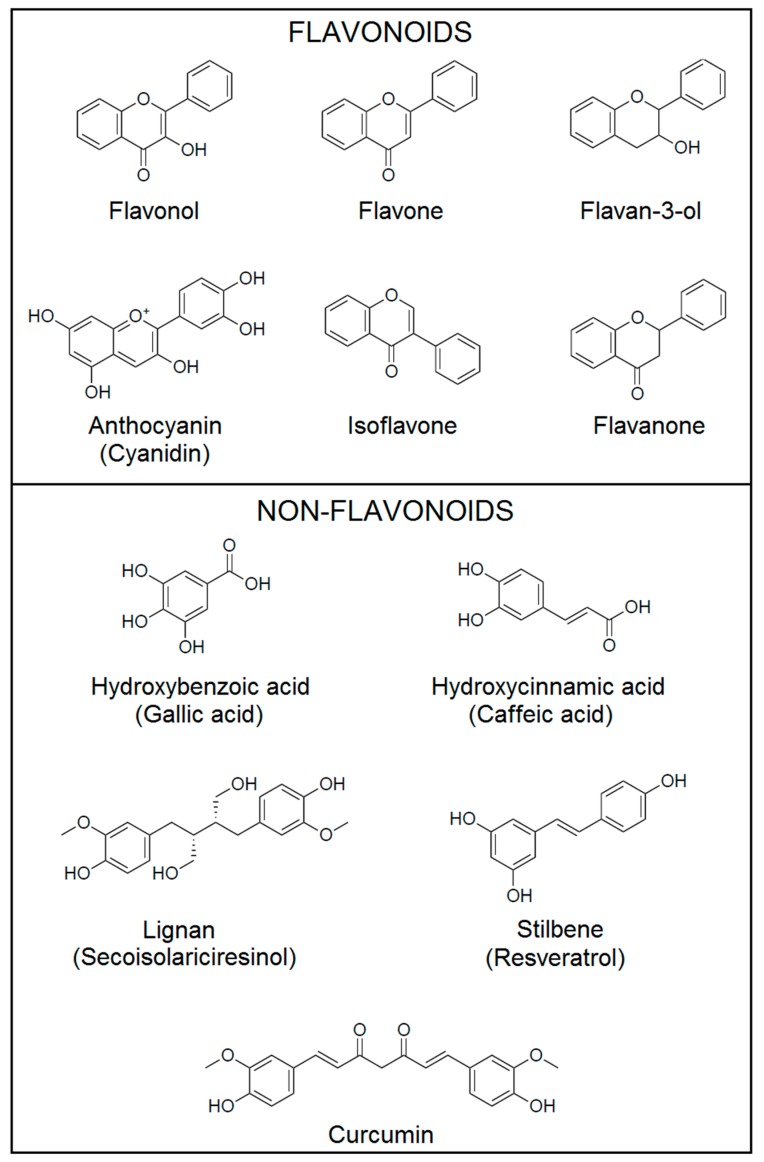
Chemical structures of polyphenols. Dietary polyphenols can be classified by their chemical structures into flavonoids and non-flavonoids. Structures generated using ChemDraw JS 17.1 (CambridgeSoft Corp., Cambridge, MA, USA).

Dietary polyphenols can be classified by their chemical structures into flavonoids and non-flavonoids (phenolic acids, stilbenes and lignans) (Figure 1) [5,6,7].

Flavonoids consist of two six carbon aromatic rings (rings A and B) connected by a 3-carbon bridge forming a heterocyclic ring (ring C), and they are further divided into different subclasses based on the different functional groups and the level of oxidation status in the ring C. The main subclasses are flavonols, flavones, flavan-3-ols, anthocyanins, flavanones and isoflavones [6]. The most abundant flavonoids in fruits, edible plants, wine and tea are flavonols, which include quercetin, kaempferol and myricetin. These compounds are mainly present in plants in glycosylated forms [3]. Flavones [apigenin (APG), luteolin, tangeretin, nobiletin, baicalein, wogonin and chrysin (CHR)] are found as 7-*O*-glycosides in parsley, celery, onion, onion leaves, garlic, pepper, chamomile tea [3]. Flavan-3-ols are found in fruits, cereals, berries, nuts, chocolate, red wine and tea. This subclass includes several compounds with different chemical structures, that can be divided in monomers, (+)-catechin, (−)-epicatechin, (+)-gallocatechin, (−)-epigallocatechin, (−)-epicatechin-3-*O*-gallate, (−)-epigallocatechin-3-*O*-gallate (EGCG) and polymers (proanthocyanidins) [3]. Anthocyanins exist in food in aglycone form (anthocyanidin) and in heteroside form (anthocyanin). Cyanidin, pelargonidin, delphinidin, peonidin, petunidin and malvidin are the most abundant anthocyanins and are present in berries, cherries, red grapes and currants [7,8]. Flavanones are mainly found in citrus fruits and they occur as aglycones [hesperetin (HSP) and naringenin], neohesperidosides (neohesperidin and naringin) and rutinosides (hesperidin and narirutin) [7]. The last flavonoids’ subclass is isoflavones, which are mainly found in soybeans, soy products and leguminous plants. They occur in food as aglycones (daidzein and genistein) and glycosides (daidzin, genistin and puerarin) [9].

Phenolic acids can be divided into hydroxybenzoic acids (protocatechuic acid and gallic acid) and hydroxycinnamic acids [caffeic acid (CA), ferulic acid, p-coumaric acid and sinapic acid]. Hydroxybenzoic acids are found in edible plants, while hydroxycinnamic acids are present in coffee, fruits and cereal grains [9].

Secoisolariciresinol, matairesinol, medioresinol, pinoresinol and lariciresinol are the most common members of lignans and are found mainly in linseed and in minor quantity in leguminous plants, cereals, algae, fruits and vegetables [7]. Resveratrol (3,5,4′-trihidroxystilbene, RES) is the main member of stilbenes and it is present in red wine, grapes, plums, berries, peanuts and pine nuts [7]. Curcumin (1,7-bis-(4-hydroxy-3-methoxyphenyl)-1,6-heptadiene-3,5-dione, CUR) is a polyphenol of the curcuminoid family and is found in the spice turmeric derived from the rhizome of Curcuma longa [9].

Polyphenols can modulate different signal transduction pathways involved in carcinogenesis in various types of cancer, both in vitro and in vivo [5,6]. Polyphenols have demonstrated a potent activity in affecting transcription factors [nuclear factor κB (NF-κB), signal transducer and activator of transcription 3 (STAT3), activator protein-1 (AP-1), β-catenin/Wnt, peroxisome proliferator activator receptor-gamma (PPAR-γ), Sonic Hedgehog (Shh), and nuclear factor erythroid 2 (Nrf2)]; growth factors receptors (EGFR, ErbB2, VEGFR, IGF1-R); protein Kinases (Ras/Raf, mTOR, PI3K, Bcr-abl and AMPK); and pro-inflammatory mediators [Tumor necrosis factor-α (TNF-α), interleukins (ILs), COX-2, 5-LOX] [10]. In addition, polyphenols possess anti-oxidant and pro-oxidant properties [6,11]. However, the in vivo beneficial effects of polyphenols are relatively poor, due to the low bioavailability of polyphenols in the human body [7,9]. It has been reported that the plasma concentration of polyphenols and total metabolites reached after an intake of 50 mg of aglycone equivalents ranged from 0 to 4 µM [12]. Indeed, polyphenols exist in food as aglycones or glycosides, and glycosylation affects absorption and then the bioavailability. The metabolism of the polyphenols in the gastrointestinal tract and liver, their binding to the microbial flora in the oral cavity and gut and to the surfaces of blood cells, the regulatory mechanisms that prevent the toxic effects of high polyphenols levels on mitochondria or other organelles, and dietary factors, are some of the mechanisms that limit the bioavailability of polyphenols [7]. In addition, to assess the in vivo potential of polyphenols, it is necessary to consider both their plasma concentration and their concentration in the target tissues. It has been estimated, in fact, that the concentration of polyphenols typically ranges from 30 to 3000 ng aglycone equivalents/g tissue in different tissues of mice and rats [1]. The evaluation of the tissue concentrations of polyphenols in humans is more difficult, and thus, the studies are very scarce [7,13].

## 3. Tumor Immune Microenvironment

The coexistence between the immune system and cancer cells is of paramount importance in the progression of the neoplastic disease. From the initial phases of the oncogenic process, when newly-formed cancer cells initiate lodging within the microenvironment, to later tissue invasion and metastatic dissemination, the immune system is involved in a paradoxical cycle of both eliminating and supporting cancer cells [14,15,16,17]. This duality is better explained by the concept of “cancer immunoediting”, that postulates the evolution of the immune system’s role from anti- to pro-tumoral in time. In the immunoediting-scale of events, the immune system has first a determinant role in eradicating emerging cancer cells (the “elimination” phase), then gradually “fading” into a longer phase in which killing of cancer cells is counterbalanced by the rise of less-immunogenic tumor clones which cannot be killed by immune mechanisms (immunoevasion) and which start to establish more active means of immunosuppression (the “equilibrium” phase). At last, the immunosuppressive mechanisms established in the equilibrium phase will both make cancer clones invulnerable to immune recognition and killing and turn immune cells into tumor-supporting cells (the “escape” phase) [14,15,16,17], henceforth allowing full cancer growth and dissemination.

### 3.1. Molecular Mechanisms of Cancer Immunoevasion and Immunosuppression

First demonstrated back in 1957 by Thomas and Burnet, the role of the immune system in clearing newly transformed cells is overtly crucial, though it comes with the backdrop of applying a constant “evolutionary pressure” to cancer cells themselves. This, in turn, selects neoplastic cells against antigenicity, leading in time to the emergence of clones which are “invisible” to the immune system or which have gained the ability to suppress immune responses (and, most often, both) [18,19]. Several mechanisms contribute to cancer immunoevasion and immunosuppression, also on different biological and temporal scales and with different “relative importance” depending on the type of tumor [19,20,21]. Here, we will review the most important mechanisms of immunoevasion and immunosuppression, as their understanding and potential exploitation is instrumental to the definition and shaping of immunotherapeutic approaches.

#### 3.1.1. Reduced Antigenicity and Immunogenicity

A most obvious and widespread route for cancer cells to avoid immunogenic cell death (ICD) is to reduce their chances of being spotted by patrolling immune cells [18]. Loss of antigenicity can either be the consequence of the selection operated by the immune system against more immunogenic cells or the result of mutations in tumor antigens or major histocompatibility complex (MHC, also known as HLA), though often the two mechanisms coexist within the heterogeneous pool of cancer cell clones [18,19,22].

Intimately linked to antigenic loss is the observed reduction in cancer cells’ immunogenicity, defined as the ability of tumor clones to blunt immune responses even if MHC molecules and antigens are expressed at sufficient levels [18]. In this context, accumulating evidence suggest that the crowd of signals on the surface of cancer cells that compete with immunostimulatory pathways for T cell activation or that are able to fine-tune immune responses are as important as antigens in predicting the potential for cancer immunoevasion. For example, the expression of galectin 9 on tumor and stromal cells is linked with the suppression of anti-tumoral Th17 responses from T cells, which are rather polarized towards an immunosuppressive regulatory T cell (Treg cells) phenotype [23]. Similarly, the appearance and the quantitative expression of negative regulators of immunity, such as PD-1, LAG-3, TIM-3, VISTA, CD244, CD160 and BTLA on the surface of intratumoral lymphocytes has important consequences for the overall response [18].

#### 3.1.2. Cytokines and Enzymatic Immunosuppression

Far beyond the reach of MHC proteins and the immunological synapses between T cells and their target cancer cells extends the immunosuppressive microenvironment of tumors, which establishes via complex reciprocal interactions between the tumor and the host [19]. It is, in fact, the highly local and heterogeneous balance of pro-and anti-inflammatory cytokines and pathways within each area of a tumor focus (encompassing cancer cells and stromal elements such as blood vessels, fibroblasts, etc., up to non-cellular components such as the extracellular matrix) [24,25] that ultimately decides the fate of the immune response. The amount and type of cytokines and other mediators involved is also dynamic in time [18,19] and an exhaustive discussion would be beyond the scope of this review. A central immunosuppressive cytokine, almost constantly involved in cancer immunosuppression regardless of the type of neoplasm, is transforming growth factor β (TGF-β), whose activities exert mainly via the SMAD2 and SMAD3 transcription factors (and their regulation) to induce suppression of inflammatory cytokines’ transcription and polarization of naïve T cells towards the Treg phenotype [14,17,19,26,27]. Almost as ubiquitous as TGF-β is the role of IL-10, the prototypical immune switch-off signal, that can suppress the differentiation and functions of practically any anti-tumoral activity [28] through mechanisms largely coinciding with that of TGF-β [29]. In addition to cytokines, several growth factors can also exert immunosuppressive actions. It is the case of colony stimulating factor (CSF)-1 and vascular endothelium growth factor (VEGF), both able to contribute to wide immunosuppression by targeting the myeloid compartment of the immune response. It is known, in fact, that the former (at least in a wider immunosuppressive context orchestrated by other tumor-derived cytokines) leads to the formation of tumor-associated macrophages (TAMs) [30,31], which are in turn key players in neoplastic microenvironmental immunosuppression [32]. The latter is conversely mostly involved in shifting the T cells balance towards Treg cells [33]; however, one of its most abundant sources of production within the tumor microenvironment is TAMs, thus linking the innate and the adaptive immune response along the immunosuppressive pathway [32]. On top of the plethora of cytokines and growth factors with confirmed or potential immunosuppressive abilities, several enzymes are also linked to cancer-derived immunosuppression. Is this the case of indoleamine 2,3-dioxygenase (IDO) and tryptophan 2,3-dioxygenase (TDO), both involved in catalyzing the degradation of tryptophan to kynurenine [19]. Not only tryptophan is essential for cell proliferation and, in its absence, T cell survival is severely compromised, but also the kynurenine generated via IDO/TDO activity exert direct immunosuppressive actions binding to the aryl hydrocarbon receptor (AhR) and leading T helper differentiation towards the Treg phenotype [34]. Similar roles are also played by arginase (ARG), an enzyme that catalyses the breakdown of arginine to ornithine thus depriving T cells of an amino acid they absolutely need for proliferation and for the generation of immunostimulating polyamines [35]. Furthermore, several membrane-integral and extracellular moieties have, beyond their intracellular signaling role, direct immunosuppressive effects whose clinical relevance are not yet fully understood. For example, various tumor gangliosides, both projecting from the cell surface and shed into the microenvironment, have potent immunomodulatory effects, mostly hinting at negative regulation of antigen presenting cells (APCs) and T helper cells [36,37].

#### 3.1.3. Suppressive Immune Cells

Treg cells, also known as CD4^+^CD25^+^FoxP3^+^ T cells, play a central role in physiological and pathological immunosuppressive reactions. Countless works show the crucial importance of tumor derived-Treg cells in cancer biology and therapy and highlight the dramatic differences with their normal counterparts, which are conversely necessary for keeping peripheral tolerance and regulating the magnitude of immune reactions [19]. In brief, tumor-derived Treg cells develop locally within or in the vicinity of the cancer focus, to which they are drawn as naïve CD4^+^ T cells by tumor-derived cytokines, and they show higher suppressive activity than physiological Treg cells, thereby suppressing other immune cells’ activity through the production of massive amounts of IL-10 (among others) or directly and indirectly killing them via Fas/FasL- or CD40/CD40L-mediated cell death or via enzymatic starvation [14,15,38,39,40,41,42]. Crucial to the generation of Treg cells are IL-2 and TGF-β, the latter particularly important as it directly induces the expression of FOXP3 (the master “identity factor” of Treg cells) via Smad factors binding to FOXP3 promoter, both of which abound in the tumor milieu [43]. In addition, further factors such as the activation of NF-kB or the JAK/STAT pathway are also deemed necessary for Treg cells development and probably concur to the high phenotypic plasticity of these cells, thus further linking Treg cells with the diverse stimuli coming from the tumor microenvironment [43,44,45]. The myeloid branch of the immune system is also heavily involved in tumor-dependent immunosuppression, with myeloid-derived suppressor cells (MDSCs), TAMs and tolerogenic dendritic cells (tDCs) all exerting key actions against the anti-tumoral immunity. MDSCs are, de facto, immature myeloid cells generated from mono-granulocytic precursors in the bone marrow under the systemic influence of constantly-produced inflammatory cytokines, as it happens in cancer. Owing to their mono-granulocytic origin, MDSCs exhibit either a polymorphonuclear (PMN, Lin^−^HLA-DR^−/lo^CD33^+^ or Lin^−^HLA-DR^−/lo^CD11b^+^CD14^−^CD15^+^CD33^+^) or a monocytic (M, CD14^+^HLA-DR^neg/lo^ or Lin^−^HLA-DR^neg/lo^CD11b^+^CD14^+^CD15^−^) phenotype, though there are no fundamental differences in their functions. These functions include arginine and cysteine deprivation, which impede T cell proliferation, production of nitric oxide (NO) and reactive oxygen species (ROS), which oxidize T cell receptors (TCRs) and cytokines leading to cell apoptosis, extensive production of immunosuppressive cytokines (primary IL-10 and TGF-β), enzymes (such as IDO) and angiogenic factors aiding both tumor dissemination and the formation of Treg cells, and a high expression of PD-1/PD-L1, receptors and ligands that are involved in direct killing of anti-tumoral T cells [46]. Similar to MDSCs, TAMs are generated under the influence of non-resolving inflammation and subsequent, constant production of inflammatory cytokines, though the effect on hematopoietic cells seems to be less important here as the majority of TAMs are likely arising from local resident macrophages rather than altered precursors [47]. At the functional level, TAMs overlap extensively with MDSCs, so that all the immunosuppressive functions already discussed are also characteristic of TAMs [47]. In fact, the two cell types are intimately linked and the differentiation of M-MDSCs into TAMs has been experimentally ascertained already [48]. It is evident that the mono-macrophagic and granulocytic tumor infiltrate, variously termed as M-MDSCs, PMN-MDSCs, TAMs or tumor-associated neutrophils (TANs) is a continuum of phenotypes [49] whose primary role is to support tumor immunosuppression and microenvironmental takeover. Much less understood are the biogenic pathways that lead to the generation of tDCs, for which, e.g., the role of relative “maturation” in determining their functions is still debated [50,51]. What is more ascertained is their immunosuppressive profile, which is linked to their ability to generate both suppressive and stimulatory cytokines at the same time, so that so-called mature immunogenic dendritic cells (DCs) produce significant amounts of IL-10 together with IL-12, TNF-α, IL-1 and IL-6. Similarly, semi-mature tDCs may still express high amounts of MHC II but lack the expression of IL-1β, IL-6, TNF-α and IL-12, while the production of IL-10 seems to be a constant throughout all the maturation stages [50,51]. In addition to a broadly adaptable cytokine profile, tDCs show complex immunogenic features, being able to efficiently present tumor-derived antigens while expressing large IDO quantities that rather kill the reactive cells [50,51].

#### 3.1.4. Exosomes

A final “layer” of immunosuppression is represented by tumor exosomes (TEXs), vesicles the size of a virus (~100 nm) carrying a cargo of RNAs and proteins with powerful immunomodulatory activity. For example, TEXs isolated from tumor cell supernatants and cancer patients showed a rich expression of FasL, PD-L1, IL-10, TGF-β as well as prostaglandin E2 (PGE2) and ectoenzymes engaged in the adenosine pathway (CD39 and D73). In addition, TEXs often carry MHC proteins, tumor antigens and co-stimulatory molecules, possibly scavenging stimulatory signals off the cancer cells and thus cooperating to immunoevasion [52]. Additionally, TEXs shuttle vast amounts of tumor-promoting RNAs such as micro-RNAs (miRNAs), which can target different processes in different cells and thus spread immunosuppression transversally through the immune system. For example, miRNAs such as miR-584, miR-517c and miR-378 target and downregulate TGF-β-associated kinase 1 (TAK1), which is necessary for the generation and function of DCs [53,54]. While mechanisms for TEX-dependent immunosuppression accumulate every day, it is becoming evident that TEXs have the potential to impair DC development, support TAM and Treg cells differentiation and to suppress the activity of natural killer (NK) cells and cytotoxic T lymphocytes (CTL) [52,54].

### 3.2. Active and Passive Immunotherapy: Basic Concepts and Mechanisms of Action

The conceptual foundation of immunotherapy, deriving directly from the immunoediting process, is simple: to regain the original anti-tumoral activity of the immune system at later stages, marshaling the specificity and long-term memory of the adaptive immune response to achieve durable tumor regression and possible cure. To this purpose, several approaches have been proposed and entered the clinical practice, turning immunotherapy from a futuristic idea to one of the most, if not the most, promising approaches in clinical oncology in just a few years [14,55]. To turn the “immune switch” on again and ensure that it stays on long enough to elicit a curative response, at least two routes can be followed: one is, pretty obviously, to infuse the patient with tumor antigens, cytokines, in vitro-activated immune cells or even cell-based vaccines to overcome the lack of activatory stimuli in the cancer microenvironment and thus re-boost the physiological anti-cancer immune activity. The other route is to remove immunosuppressive mechanisms established by cancer cells, re-enabling the activation of intra- and peri-tumoral immune cells and the eradication of the neoplasm. Moreover, looking at the mechanisms by which immunity is activated or immunosuppression is removed, immunotherapy protocols can be further subdivided into “active” and “passive” categories. As the names suggest, active immunotherapy seeks the induction of a de novo curative response in patients, in the absence of a pre-existing response against the given neoplasm (or better still, against the given tumor-associated antigen, TAA), while passive immunotherapy transfers pre-formed immune system components of the anti-tumoral response into patients and/or removes the blocks that prevent it.

#### 3.2.1. Therapeutic Cancer Vaccines

Despite fundamental biological differences, all therapeutic cancer vaccines share the same goal: to increase the load of TAAs available for immune recognition, inducing the expansion of tumor-specific curative T- and B-cell clones able to recognize and destroy cancer cells [55,56,57,58]. To this aim, a wide variety of vaccines, based on proteins, genes and cells have been proposed.

##### Proteins/Peptides Vaccines

A most intuitive way to achieve such a goal is to purify TAA proteins/peptides in the laboratory, either from the patient’s tumor itself or from suitable cell lines recapitulating patient’s neoplasm, reproduce them in large quantity, mix with an adjuvant or immune modulator to enhance the immune response to the TAAs and then inject them back into the patient. While simple in theory, practice has proven difficult for this approach for many reasons. First, not all TAAs are equally present on cancer cells’ surface nor they are equally important for cancer functions, making the identification of candidate TAAs difficult [14,16,55,56,57]. Second, these vaccines target only one or a few epitopes, making for short peptide products to be injected.

##### Genetic Vaccines

Unlike the proteins/peptides approach, genetic vaccines (either DNA- or RNA-based) can deliver large chunks of antigenic information into target cells, making it possible to induce the expression of multiple TAAs with a single immunization [38,55,57,58,59]. Furthermore, with a suitable choice of a genetic vaccine and a vector, it is possible to induce ectopic TAAs expression not only on cancer cells, but also, and possibly concomitantly, on the DCs which are needed to elicit complete T cell activation [55,60,61], thus resulting in cross-priming and/or direct antigen presentation and large benefits in terms of response magnitude.

##### Cell-Based Vaccines

In the context of installing a de novo response to cancer, cell-based vaccines have until now shown a great potential [16,17,38]. Basically, a cell-based vaccine relies on loading DCs with TAAs in vitro, after which the activated DCs are injected into the patient. When homed to secondary lymphoid organs, the DCs will present TAA epitopes to both CD4^+^ and CD8^+^ T cells and induce long-lasting anti-tumoral immunity [38,55,62]. Further means of stimulating the immune response, such as the co-injection of (or the co-transfection of DCs with) adjuvants, cytokines and co-stimulatory molecules have been and are, also, actively studied in clinical trials [55,62,63].

#### 3.2.2. Cytokine Therapy

The oldest approach to immunotherapy belongs to the group of passive protocols. Rather than the development of a de novo response, in fact, cytokine therapy seeks to either exert cytotoxic effects on cancer cells (thus making up for the insufficient response of the patient’s immunity) or to indirectly enhance the patient’s anti-tumoral response by globally boosting the activity of his or her immune system. Research on cytokine treatments is intimately linked to basic research on immunology and cancer immunobiology, and, unlike any other immunotherapeutic protocols, can rely on an enormous “corpus” of knowledge and more than two decades of active research [38,64]. Still, the complexity and the integration of the immune responses at the systems’ level, the pleiotropic effects of all cytokines, the presence of physiological negative feedback mechanisms tampering with cytokine activity, the ability of tumor cells themselves to produce and utilize cytokines to their own advantage and, most importantly, the dramatic presence of side effects has significantly reduced the number of cytokines that have been approved for oncological treatment [38,55,64]. As a result, since the early 1990s, only a few immunostimulating cytokines [IL-2 and interferons (IFNs), mostly α and β, though also γ has found limited applications] have made it to the bedside. Yet, research on cytokines for immunotherapy is far from being over: from IL-2-family cytokines to pegylated, long-lasting cytokines and from virally-delivered cytokines to antibody-cytokine fusion molecules [38,55,64], there are many promising approaches involving cytokine therapy.

#### 3.2.3. Co-Stimulatory Receptors Therapy

Conceptually similar to cytokine therapy is co-stimulatory receptors therapy. The basic principle of this approach postulates that the overall activity of patient’s lymphocytes (including anti-tumoral clones) can be regulated via manipulation of the co-stimulatory signals that are crucial to the normal activation and polarization of T cells [14,16,38]. Hence, recombinant molecules (most often antibodies) able to induce co-stimulatory signaling in T cells show promising anti-tumoral effects, especially when used in combination with other immunotherapeutic approaches. Furthermore, much like cytokines, co-stimulation is not necessarily restricted to targeting T cells. Co-stimulatory signals are, in fact, typically expressed by APCs upon their commitment to antigen presentation and presented to the T cells together with TAAs. It follows, then, that agonists are able to fully activate APCs, thus inducing up-regulation of their co-stimulatory signals, can serve the role of “indirect” co-stimulators. Toll-like receptor (TLRs) agonists are a good example: typically pro-inflammatory agents of microbial origin, TLR agonists fully activate APCs inducing massive expression of the co-stimulatory molecules CD80 and CD86 and these, in turn, convey together with the TAAs the necessary set of instructions for the activation and expansion of anti-tumoral T cell clones [55].

#### 3.2.4. Adoptive T Cell Transfer Therapy

The identification, more than 20 years ago, of tumor-infiltrating lymphocytes (TILs) showing selective activity against the tumors from which they have been isolated has not only laid the foundation of immunoediting (providing proofs of the elimination phase in action), but in all accounts has sparkled the very idea of immunotherapy [14,15,16,17]. Nowadays, three main approaches have made it to clinical trials, each with its own advantages and disadvantages. Despite large biological and technical differences, all are based on the purification of T cells from the patients followed by in vitro activation or genetic manipulation and re-injection into the patients.

##### TILs

The isolation and reinfusion (hence, the transfer) of autologous T cells within the same patient is a passive protocol offering the enormous advantage of a truly personalized medicine approach which, thanks to the in vitro manipulation, conjugates high efficacy to complete immunocompatibility [41,65]. Unfortunately, while a sound golden standard in theory, the adoptive transfer of TILs is far from being a viable option for most cancers. At first, in fact, one must acknowledge that while TILs are ubiquitous in cancer foci, their net mass varies significantly between the different neoplasms, making the isolation process relatively easy in some cases while extremely cumbersome in (many) other [55,66]. In addition, while in vitro pulsing with cytokines or other immunostimulating agents is virtually safe and free from side effects at reinfusion, “supporting” cytokines must currently be administered at transfer too, in order to expand and keep the TILs active in vivo, with all the side effects that it implies. Intrinsically linked to the need for supporting cytokines is the rapid decline of infused TILs’ functions in vivo, most likely due to the intratumoral presence of immunosuppressive Treg cells.

##### TCRs

As the most obvious limitation to a wider use of TILs in cancer immunotherapy is their typical very low frequency, several approaches have been developed using molecular biology methods to “create TILs” in vitro. All these approaches relate to some degree of engineering of the TCR, and are generally passive approaches, though some cases are borderline to the active immunotherapy [67]. In the simplest case, a rare TIL population is isolated from a patient, the α and β TCR chains cloned into a vector and transduced into an autologous population of naïve T cells as to “transform” all the recipients into TILs, whose number can be further expanded in vitro before reinfusion [67]. The TCR sequence can of course be further modified in vitro, for example by increasing its avidity [68], before clonal expansion and re-injection. Unfortunately, the same limitations that apply to TILs apply to such an approach as well: the need for a pre-existing coding sequence recognizing the tumor restricts the material from which to start to impractically low numbers. As an alternative to circumvent the need for a starting sequence, mice engineered to express human HLA system can be injected with tumor derived proteins, to which they will react mounting a specific antitumoral response led by T cell immunocompatible to humans (which will then be isolated and used to clone the TCR into human T cells) [67]. In a similar way, heterologous T cell gene transfer can be pursued, that postulates the isolation of antitumoral T cell clones from a patient in remission, cloning of their TCRs and the transfections of naïve T cells of a second patient with the same neoplasm but experiencing no remission [67]. Finally, the most recent incarnation of adoptive T cell transfer extends beyond engineering TCR itself, rather creating an entirely new form of receptor called, appropriately, chimeric antigen receptor (CAR) [67,69]. In CARs, the antigen-binding region is in fact derived from an antibody (typically a single-chain fragment variant, the auto-assembly of the antigen-binding regions of antibodies’ heavy and light chains), with significant gains in the type and size of antigens recognized and without HLA-restriction

#### 3.2.5. Immune Checkpoint Inhibitors

To ensure the possibility to regulate the magnitude and the extension, in space and time, of immune activity as well as to guarantee self-tolerance and prevent autoimmunity, a complex network of inhibitory pathways exists within immune cells with the aim to suppress cell activation [67,70]. These pathways are collectively referred to as “immune system checkpoints”, and their activity is mainly directed towards the shutdown of T cell activation or effector functions. In recent years, it has become clear that a major mechanism of tumor immunoevasion is to leverage on such checkpoints to remove anti-tumoral T cell clones from the microenvironment, and this has in turn spurred an outgrowth of passive immunotherapeutic approaches aiming at removing the engagement of the checkpoints operated by tumors [67,70].

##### CTLA-4

Cytotoxic T-lymphocyte antigen-4 (CTLA4, also known as CD152) is a well-characterized inhibitory co-receptor expressed by both helper and cytotoxic T cells, whose role is to restrain T cell activation by competing with the activatory co-receptor CD28 for binding to the co-stimulatory molecule CD86 expressed by APCs. CTLA-4 has greater affinity for CD86, so it rapidly outpaces CD28 in competitive binding; on the other hand, while CD28 expression on T cell surface is constitutive, CTLA-4 is primarily an intracellular antigen whose shuttling and permanence on the cell surface is tightly controlled [55,67,71]. Ultimately, then, it is the balancing between dynamics and affinities of the two antagonistic co-receptors that governs the initial activation and the later shutdown of the T cell functions in the lymphatic tissues [28,31]. Genetic depletion of CTLA-4 in mice leads to a lethal phenotype due to deleterious systemic immune hyperactivation [72], but also shows that impeding the functions of this co-receptor leads to a drastic enhancement of the immune functions, which can then be exploited for cancer immunotherapy. Currently, recombinant antibodies that target and block the function of CTLA-4 have in fact shown promising results against various solid cancers [67,70].

##### PD-1/PD-L1

Programmed death-1 and its ligand (PD-1 and PD-L1, respectively) are immune checkpoint co-receptors with a wide expression across the lymphoid branch and with a primary function in peripheral tissues rather than in lymphoid organs (unlike CTLA-4), tasked with inducing anergy and, later, T cell death upon engagement. Being ubiquitously expressed by T cells during the course of an immune reaction [73], PD-1 is easily targeted by cancer cells, which frequently overexpress the PD-1 ligands 1 and 2 (PD-L1 and PD-L2) [73]. The wider expression of PD-1 and the milder autoimmune reactions observed in animal models of genetic *Pdl*, *Pdl1* or *Pdl2* ablation in respect to CTLA-4 [55,63,73] make these molecules attractive candidates to immunotherapy. In fact, various recombinant antibodies targeting either PD-1 or its ligands are now under active development and tested for clinical use in different cancers [74].

##### Treg cells

The concept of removing immune checkpoints can be, finally, expanded to those cells (primarily the Treg cells) whose role is to suppress immune functions by inhibiting lymphoid activation [40]. In cancer foci, Treg cells tend to appear in tune with the oncogenic process and, behaving as the immunosuppressive counterpart to TILs, they get activated by TAAs and install suppression of anti-tumoral TILs [40,55]. Targeting Treg cells within the cancer microenvironment is, then, another possible approach to liberate infiltrating T cells and allow for their reactivation. In this context, various approaches primarily aimed at stimulating TILs, such as anti-CTLA-4 antibodies or TLR agonists, also work at inhibiting Treg cells, thus opening up interesting possibilities for combined immunotherapy approaches [40,70]. Unfortunately, Treg cells show a high heterogeneity, whose clinical importance is far from being understood, and different Treg cells subtypes can, depending on the approach, show complete and sometimes paradoxical reactions (such as the depletion of some clones and the activation of other) [42].

## 4. Polyphenols and Immune Cells Modulation

**Figure 2:**
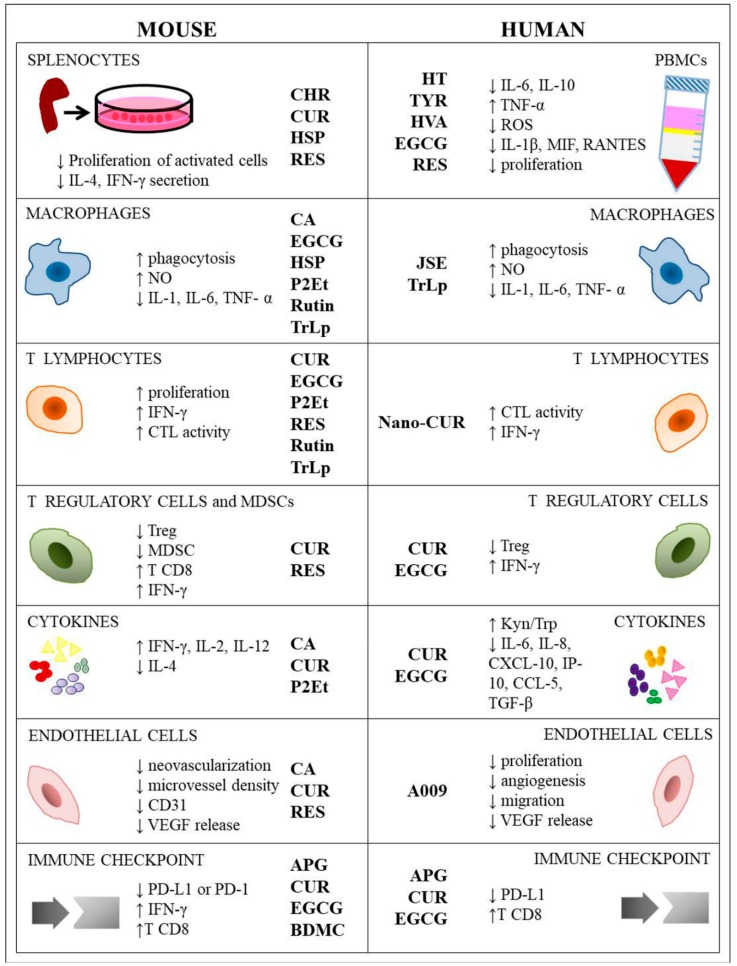
Effects of polyphenols on immune cells. The more common activities of polyphenols on immune cells and cytokines are reported.

The effects of polyphenols on immune response are summarized in Table 1 and Figure 2.

### 4.1. Peripheral Blood Mononuclear Cell (PBMCs) and Murine Splenocytes

Various studies have demonstrated the anti-inflammatory effects of polyphenols on total human PBMCs or murine splenocytes. Though unspecific, these studies are informative as they provide a global overview of the potential of polyphenols as chemopreventive molecules, able to reduce local inflammatory responses and, thus, diminish the risk of further pathological tissue manifestations and neoplastic conversion.

For instance, Serra et al. evaluated the activity of olive oil phenolic compounds [such as hydroxytyrosol (HT), tyrosol (TYR) and homovanillic alcohol (HVA)] on human PBMCs stimulated with oxysterols, derived from cholesterol oxidation. Results showed that pre-treating PBMCs with olive oil’s phenols reduced the secretion of pro-inflammatory cytokines/chemokines (IL-1β, RANTES) and decreased intracellular ROS content [75], therefore diminishing the contribution to an inflamed microenvironment, known to facilitate tumor onset. Importantly, Soto et al. showed different and dose-dependent in vitro and in vivo effects of another polyphenol, RES. In vitro, RES induced a dose-dependent reduction of NXS2 (murine neuroblastoma) and M21 (human melanoma) cell proliferation, mostly due to cytostatic rather than cytotoxic events, that was mirrored by an anti-proliferative effect on mouse splenocytes and human PBMCs induced to proliferate with Concanavalin A (ConA) and Phytohemagglutinin (PHA)/IL-2, respectively, and by reduced ability of effector cells to lyse target cancer cells by antibody dependent cellular cytotoxicity (ADCC). Conversely, in vivo studies confirmed the anti-tumoral activity of RES on the same neuroblastoma cells but also highlighted the lack of immunosuppressive effects (both at the blood counts’ level and for what concerns the activity of isolated peritoneal macrophages) at a dose of 50 mg/kg, and they rather evidenced increased leukocyte counts in the tumor microenvironment [76]. In line with these findings, RES was combined with IL-2 for in vivo treatment and found that, in mice treated with RES/IL-2, all tumors regressed and very infrequently recurred or metastasized. Accordingly, the survival at day 100 post-inoculum of tumor cells was 61% for mice in this group as compared to 15% in RES group and 13% in IL-2 group, suggesting that the immunomodulatory effects of RES are dose-dependent and that polyphenols in general can elicit strong chemo-supportive effects [76]. On the other hand, a study by Chang et al. confirmed that other polyphenols such as linalool and p-coumaric acid inhibited the proliferation of A549, T-47D, SW-620 and Hep G2 cell lines in a dose-dependent manner, though they significantly stimulated the production of pro-inflammatory cytokines such as CD40-ligand, CD40, IFN-γ, IL-12 p40, IL-13, IL-17F, IL-1β, IL-2, IL-21, IL-21R, IL-23p19, IL-4, IL-6sR and TNF-α, when used to stimulate lymphocytes from healthy donors [77]. The duality of anti- and pro-inflammatory effects of different polyphenols is also evident in the works by Sassi et al. [78]. In one study, it was found that HSP (3′,5,7-trihydroxy-4′-methoxyflavone) reduced the proliferation of LPS-stimulated splenocytes from male Wistar rats, while it over-stimulated the proliferation of the same cells pulsed with lectin, though with no effect on CTL activity or NO production by macrophages [78]. On the other hand, a similar polyphenol (5,7-dihydroxyflavone, CHR) gave quite opposite effects in the same experimental condition. CHR, in fact, inhibited the proliferation of LPS-stimulated splenocytes, but inhibited lectin-stimulated cells even stronger, at the same time increasing CTL activity and macrophage lysosomal activity, yet inhibiting the production of NO in unstimulated macrophages [78]. Gao et al. demonstrated that RES has a dose-related effect on ConA-stimulated splenic lymphocytes; in fact, proliferation was suppressed at doses higher than 25 µM while it was significantly increased at lower ones. Intra gastric administration of RES did not changed proliferative response in spleen of C3H mice nor was able to change IFN-γ production, demonstrating a discrepant behavior towards immune cells in vitro or in vivo [79]. Finally, recent evidence on EGCG suggest a significant anti-proliferative and immunosuppressive effect on PBMCs isolated from newly diagnosed breast cancer patients or age matched controls stimulated with PHA, anti-CD3, or Her2/neu and p53 antigen peptides [80], further implying that polyphenol effects in vivo are compound-, dose- and cell-specific.

### 4.2. Macrophages

Several polyphenols have been shown to stimulate the proliferation as well as the phagocytic activity of macrophages, resulting in a reduction of tumor proliferation or tumor volume in in vitro and in vivo models; however, in this case, contrasting results also exist.

Huang et al. demonstrated that the EGCG promoted and stimulated Mac-3 expression and phagocytic activity by macrophages isolated from PBMCs of leukemic Balb/c mice, intraperitoneally injected with murine leukemic WEHI-3 cells [81]. Studying the same molecule, Jang et al. discovered that EGCG-treated 4T1 tumors in Balb/c mice showed a diminished level of chemokines (CSF-1, CCL-2) and a reduced infiltration of M2 macrophages and TAM. These cells had a cytokine/chemokine profile (IL-6 low, TGF-β low) that suggested the switch towards an M1 phenotype (TNF-α high) obtained with influence of TEXs after EGCG treatment [82]. Similarly, Lin et al. demonstrated that rutin (a glycoside combining the flavonol quercetin and the disaccharide rutinose) stimulated phagocytic activity in macrophages derived from PBMCs or collected from the peritoneal cavity [83]. Furthermore, Orsolic et al. evaluated the effect of CA in mice, injected with Ehrlich ascites tumor (EAT) cells. The compound reduced tumor cell growth and ascitic fluid volume as well as it increased the mean survival time. Notably, CA was effective in increasing macrophage counts in the peritoneal cavity as compared to control, while neutrophils and lymphocytes were not affected. In addition, the macrophage cytotoxic potential against EAT cells was increased, while diminishing ARG1 activity without any appreciable effect on NO production as compared to control [84]. Furthermore, Alonso-Castro et al. demonstrated the safety and the immune-stimulant effect of the ethanol extract of Justicia spicigera leaves (JSE), containing kaempherol-3,7-bisrhamnoside (kaempferitrin, KM). The compound, in fact, strongly potentiated the phagocytic ability of human macrophages, differentiated from PBMCs, against Saccharomyces cerevisiae, inducing in a dose dependent manner phagocytosis, NO production and H_2_O_2_ release while also stimulating the proliferation and NK cell activity in murine splenocytes [85]. Finally, Mukhereej et al. have recently formulated a new compound (TriCurin, TrLp), where CUR is encapsulated in liposomes together with epicatechin gallate (E) and RES at a specific ratio (TrLp is CUR:E:RES = 4:1:12.5). In mice challenged both intra-cranially with GL261 mouse glioblastoma cells and subcutaneously with HPV^+^ TC-1 cells, TrLp reduced the tumor growth, also modifying the phenotype of TAMs that switched from anti- (M2, ARG1^high^iNOS^low^IL-12^low^IL-10^high^) to pro-inflammatory (M1, ARG1^low^iNOS^high^IL-12^high^IL-10^low^). Furthermore, in both models, TriCurin suppressed phosphorylated (p)-STAT-3 and induced p-STAT1 and NF-κB, which consequently induced iNOS and produced NO, toxic for tumor cells [86,87].

Contrasting with the immune-stimulatory effects of polyphenols, Sharma et al. reported on the suppression of immune responses in LPS-activated macrophages, with pro-inflammatory (IL-1, IL-6 and TNF-α) cytokine production reduced after treatment with either RES or CUR while IL-10 was increased. In addition, both RES and CUR diminished the expression of CD80 and CD86 without modification of CD40 expression [88]. Furthermore, Noh et al. demonstrated that RES acted, in vitro, suppressing the IFN-γ-induced activity of indoleamine-2,3-dioxygenase (IDO) needed for proliferation of primed-antigen-specific CD8^+^ T cells. In fact, RES abolished the expression of interferon regulatory factor (IRF)-1, which is an essential transcription factor for IFN-γ-induced IDO expression, through a signaling cascade involving the suppression of STAT-1 and protein kinase C (PKC)-δ [89]. Finally, in addition to these findings, possible biphasic behaviors of polyphenols (affected by doses of compounds or by the activation status of cells) must be considered. Gualdoni et al. showed that RES stimulates, rather than suppresses, IDO enzyme in healthy humans, as demonstrated by the slight increase in kynurenine (Kyn) and the great decline in tryptophan (Trp) levels observed at 2.5 and 5 h after RES treatment. Therefore, the behavior of IDO seems to vary according to the redox status of the milieu: in an unstimulated environment, RES increases IDO activity while in conditions of ongoing immune stimulation the compound inhibits IDO and inflammatory signals [90].

Overall, analysis of the effects of polyphenols on APCs showed to be beneficial, since the majority of data collected [78,81,83,84,85,86,87,91], point to an increased proliferation and an augmented phagocytosis activity of these cells fundamental for the following steps of the immune response.

### 4.3. T Cells

The effects that polyphenols exert on the activity of macrophages naturally lead to the analysis of T cell functions, being macrophages professional APCs stimulating T cells. As before, contrasting reports can be found, though in this case, most of the research seems to point to an immunostimulating function of polyphenols.

Lasso et al. tested gallotannin-rich standardized fraction (P2Et) from Caesalpinia spinosa, containing a high proportion of galloylquinic acid derivatives and pentagalloylglucose and lower proportion of gallic acid-containing compounds (gallates) on healthy C57BL/6 mice. It was found that P2Et increased the number of activated CD8^+^ and CD4^+^ cells as well as CD69^+^ NK, and regulatory CTLA4^+^Foxp3^+^ Treg cells. DCs and MDSC-like cells were amplified in the spleen of treated animals. At the same time, augmented serum concentrations of IL-10, IL-17, IFN-γ, IL-6, IL-4 and IL-2 was detected in the treated group. To take into consideration a possible strain specific response to P2Et, the same analyses were applied to healthy BALB/c mice and the results were overlapping except for cytokines levels, as only IFN-γ and IL-6 were found increased in serum [91]. However, in a more complex environment, such as that of tumors, specific polyphenol effects on T cells might be poorly relevant, while these compounds could still strongly stimulate immunity through primary effects on cancer cells. A clear demonstration of such “hierarchy of effects” is brought by Gomex Cadena et al., who demonstrated that P2Et induces ICD in B16F10 mouse melanoma cells via the mitochondrial intrinsic pathway. In this model, in fact, P2Et induced apoptosis but, most importantly, target cells strongly expressed those danger-associated molecular patters (DAMPs) recognized to be molecular markers of ICD, such as ecto-calreticulin (CRT), ATP and high-mobility group box 1 (HMGB1). As a proof of increased immunogenicity, as the founding reason for melanoma cell death, P2Et-treated cells were injected in C57BL/6 mice, showing that they worked as a cell vaccine delaying further tumor growth. Splenic activated (CD44^+^) and central memory (CD62L^+^CD44^+^) CD8^+^ T cells levels were also found to have increased. In parallel, in vivo treatment with P2Et increased the frequency of spleen conventional DCs (CD45^+^CD220^−^CD11c^+^) and their expression of co-stimulatory molecules (CD86, CD40, MHCII, CD70). Moreover, the phagocytic ability of bone marrow dendritic cells (BMDCs) against B16F10 cells in vitro was increased [92]. Other authors observed an increase in T cells after in vitro or in vivo administration of polyphenols. Lin et al. have demonstrated that rutin, in addition to being a strong agent for tumor reduction in the WEHI-3-induced leukemia model, also affects the level of CD3 and CD19 that were found increased in the blood after 3 weeks of treatment. Conversely, CD11b and Mac3 were found decreased [83]. The increased expression of CD19 and CD3 was also accompanied by increased cytotoxic activity of NK cells in the same murine model of leukemia treated with EGCG, though in this case, the frequency of CD11b was not influenced [81]. Furthermore, Chen at al. investigated the effects of RES on renal carcinoma microenvironment and found increased T cell-dependent activity. A switch was observed in the expression of T-helper (Th) 2 cytokines, such as IL-6 and IL-10, to Th1 cytokines with dominance of IFN-γ, and increased the infiltration of tissues from activated CD69^+^ CD8^+^ T cells, as shown with flow cytometric and immunohistochemistry analysis. In addition, the cytotoxicity was tested through mRNA expression of perforin, granzyme B and FasL, and all of them were found increased as compared to control, thus matching with the concomitant increase in the expression of Fas on tumor cells [93]. Milano et al. determined that a preparation of nano-CUR reduced the secretion of anti-inflammatory cytokines (TNF-α, IL-8, IL-6, IL-10, IL-1β) in activated T cells, but neither changed phenotype and basal level of cytokine production in resting T cells nor modified frequency of activated CD4^+^ and CD8^+^ T cells [94]. Lu et al., who developed an intracellular-labile amphiphilic CUR-based micelles delivery system (CUR-PEG), observed a highly significant inhibition of tumor growth matched with a strong CTL response and high amounts of IFN-γ when this polyphenol micelles were administered in combination with lipid/calcium/phosphate (LCP) Trp2-based vaccine to treat B16F10 advanced melanoma in C57BL/6 mice [95]. Starting by the knowledge that autophagy is exploited either by tumor cells or by healthy cells to front unfavorable conditions, such as drug assault [96], the combination of CUR with the autophagy inhibitor Chloroquine (CQ) was evaluated. When administered with CUR, it failed to support antitumor effect of polyphenol compared to CUR alone and opposite to in vitro behavior. An increase in CD8^+^ T cell and a decrease of Foxp3^+^ Treg cells were detected in peritumoral area of HER2/neu^+^ TUBO-transplanted immunocompetent Balb/c mice treated with CUR. This finding confirms the involvement of immune cells to contrast tumor growth and the benefit given by polyphenol in the tumor microenvironment and the adverse effect of CQ on immune cells [97]. Liao et al. evaluated the effect of CUR treatment, in vitro and in vivo, on oral squamous cell carcinoma cell lines, CAL-27 and FaDu, and on 4-nitroquinoline-oxide-induced (4NQO) mouse model. In agreement with many other findings [98,99], the authors confirmed the ability of CUR to inhibit tumor cell proliferation in vitro and tumor growth in vivo, but they also found an increase in CD8^+^ T cells in the tumor microenvironment which correlated with delayed tumor growth [100]. Luo et al. provided demonstration that delay in tumor growth and prolonged survival, after CUR treatment in LLC-tumor bearing mice, were achieved with the contribution of T cells. In fact, when nude mice were transplanted with tumor cells, CUR lost its effect against tumor growth. Low-dose CUR increased frequency of CD4^+^ and CD8^+^ T lymphocytes, in spleens of immunocompetent tumor-bearing mice. Moreover, CD8^+^ T cells showed a significant production of IFN-γ and an enhanced proliferation of LLC-specific CTL cells after CUR treatment [101].

Opposite results were provided by Sharma et al., who evaluated the effect of RES and CUR on lymphocytes from healthy Balb/c mice and found that, when lymphocytes were stimulated with mitogen (ConA), a dose-dependent reduction of proliferation of T cells was registered together with a diminished production of IFN-γ and IL-4, with either both compounds alone or their combination. Furthermore, both compounds decreased the expression of CD28, but while CUR also promoted CTLA-4 expression, RES lacked effects on it [88]. Kim et al. reported the failure in the completion of T cell activation after priming with CUR-treated DCs. In fact, CUR influenced the maturation of BMDCs, through the downregulation both of specific markers (CD80, CD86, MHCII) and cytokines (IL-1, IL-6, TNF-α). The defect of costimulatory and signaling molecules implicate an impaired antigen presentation that lead to insufficient IFN-γ production by T cells [102]. On a similar note, Lasso et al. showed that in vivo treatment with P2Et was able to reduce tumor growth and metastasis in transplantable models of melanoma (B16) and breast cancer (4T1), although prophylactic pre-conditioning with P2Et (see above), though theoretically anti-tumoral, not only abrogated the effects but was also detrimental. At the basis of such dichotomy is the effect of double stimulation with P2Et: while a single dose is, in fact, able to induce proliferation of CD4^+^ and CD8^+^ T cells, the double treatment (pre-conditioning + post-inoculum administration) increased CD4^+^ naïve cells (TN, CD45RB^+^CD62L^+^), but reduced the frequency of CD4^+^ effector memory T cells (TEM, CD45RB^−^CD62L^−^); the same trend was observed for CD8^+^ T cells [91].

Although these last reports show seemingly minor detrimental effects, the finding that polyphenols possess favorable properties is common. This is particularly true for the ability of polyphenols to increase CD8^+^ T cells frequency and their cytotoxic activation and ability to produce cytokines [78,86,89,91,92,93,94,97,100,101,103]. However, observation in vitro were later transferred in vivo and contrasting results were often reported [76,97]. This scenario emphasizes the complexity of the immune system.

### 4.4. Treg Cells and MDSCs

As for the effector branch of lymphoid and myeloid cells, polyphenols also show contrasting effects on immunosuppressive cells.

When administered to healthy subject, RES showed an influence on immune cells. Espinoza et al. demonstrated a significant increase of circulating Treg cells (CD3^+^CD4^+^CD25^+^CD127^dim/neg^) after 4 weeks administration of RES (1 g/day) on a healthy individual. In the analyzed blood sample, the increase was also induced in γδ^+^ NKG2D^+^ T cell and CD3^−^CD56^+^ NKG2D^+^ NK, while CD8^+^ and CD4^+^ T cells and CD19^+^ B cells did not change. Measurement of cytokines production resulted in a significant decrease of TNF-α and MCP-1 in the RES treated group, maintained for 2 weeks after interruption of RES administration. In vitro culture of Treg cells in presence of cytokines, TCR stimulation and RES conferred to suppressive cells a stronger stimulus for proliferation, which was absent when TCR stimulation was lacking [104].

Liao et al. found a systemic reduction of (CD4^+^CD25^+^Foxp3^+^) Treg cells and an increase of CD8^+^ T cells in the peripheral blood and lymph nodes of mice bearing induced 4NQO oral squamous carcinoma and treated with CUR, as they also observed a significant reduction of (CD11b^+^GR1^+^) MDSCs in these mice [100]. Similarly, Lu et al. observed a net decrease in the frequency of MDSC cells in C57BL/6 mice challenged with B16F10 melanoma cell line and treated with CUR-PEG and LCP Trp2-based vaccine, matched by a significant decrease in Treg cells and a significant increase in CD8^+^ T cells [95]. Along with these findings also those from Liu et al. in CUR-treated LLC lung cancer model reached same conclusions. CUR given to LLC-bearing mice determined a reduction of MDSC among CD45^+^ cells both in spleen and in tumor masses. In tumor infiltrating MDSCs, a decrease in suppressive-activity characterizing factors (ARG1, iNOS, ROS) and an increase in maturation markers (F4/80, MHCII, CD80, CD11c) were observed. An increased frequency of CD4^+^ and CD8^+^ T cells and a decreased level of IL-6 in tumor tissue were also found [105].

Two different publications demonstrated that CUR converts CD4^+^CD25^+^Foxp3^+^ Treg cells into IFN-γ-producing Th1 cells. Zou et al. studied lung cancer patients treated 2 weeks with CUR (3 g/day) or placebo and compared them to healthy donors, and thereby confirmed the results ex vivo culturing PBMCs from lung cancer patients with CUR [106]. Xu et al. demonstrated the same plasticity in advanced colon carcinoma patients treated, after surgical removal of tumor, with CUR (3 g/day for 1 month). The frequency of Treg cells, after therapy, was brought back to a low level, similar to healthy subjects; conversely, (CD4^+^CD25^+^Foxp3^−^) T effector cells were increased, and further investigation revealed the ability of these cells to produce IFN-γ (Th1), instead of IL-4 (Th2). ChIP analysis confirmed that CUR changed Foxp3 promoter locus suppressing its transcription in favor of IFN-γ expression in CD4^+^ T cells [107].

A clinical trial evaluated the effect of 6-month orally administered EGCG to chronic lymphocytic leukemia (CLL) patients Rai stage 0, that did not undergo therapy but waited for the evolution of disease (“wait and see”). 80% of patients that completed the study showed a reduction of circulating lymphocytes and Treg cells, usually increased in CLL, together with a drop of IL-10 and TGF-β serum levels; therefore, indicating a possibility of influence on circulating cells in CLL patients [108].

Furthermore, Chen et al. showed that RES administration decreased the frequency of Treg cells in mice transplanted with renal cell carcinomas, while that of MDSCs was increased at the higher dose of treatment though to a non-significant level [93]. All these findings are in line with previous studies from Yang et al., who analyzed the effect of RES treatment on the dynamics of Treg cells ex vivo. Spleens of EG7 (syngeneic lymphoma)-bearing C57BL/6 mice were collected and analyzed 20 days after tumor inoculation and a dose-dependent decrease in the ratio of Treg cells (CD4^+^CD25^+^)/total CD4^+^ and in the number of FoxP3^+^ cells among CD4^+^CD25^+^ Treg cells was detected as a consequence of RES treatment. This profile was also confirmed by the ex vivo analysis of spleens of Balb/c mice injected with colon carcinoma CT26 line and, in vivo, by the effects of a single i.p. injection of RES in EG7-bearing mice. In the latter case, in fact, a reduction of Treg cells frequency was again detected, together with a reduction in TGF-β production and an increase in IFN-γ produced by intranodal CD8^+^ T cells [103].

However, in contrast with the above results, double treatment with P2Et (prophylactic plus therapeutic, see above) resulted in an increased proportion of tumor-infiltrating MDSC-like cells both in melanoma and in breast cancer in vivo models [91]. Additionally, Sharma et al. evaluated the effects of RES or CUR on ConA-stimulated lymphocytes of healthy Balb/c mice, reporting absence of variation in Treg cells phenotype and frequency [88]. As for the effector branch of immunity, then, these results suggest complex and diverse regulatory mechanisms of polyphenols, which are intertwined with the status of cell activation, the type and magnitude of the stimulus and the dose and type of the phenolic compounds, and urge researchers to avoid conceptual simplifications.

Reduction in Treg cells and/or MDSCs are, in several cases [93,95,100,103,105,106,108], effects shared by polyphenols treatment, although some apparently contradictory results have been shown. Such contrasting data are found in literature because investigations were performed in different scenarios and focusing on different immunological cell subtypes, molecules or cancerous histotypes, along with different concentrations or procedures in treatment or administration that preclude a perfectly fitting comparison. In our view, the reduction of Treg cells together with increased APC functions and CD8^+^ T cells activity contribute to the establishment of the appropriate environment to reach the eradication of tumors and are therefore desirable outcomes of polyphenol treatments.

### 4.5. Cytokines

The analysis of cytokines secretion in several settings reported an increase in pro-inflammatory cytokines (e.g., TNF-α, IFN-γ) and a decrease in others because of polyphenol administration. Doubts still remain on what the real cells of origin of these cytokines are, since ex vivo assays on the complex tumor microenvironment are not specific enough to discriminate whether they derive from immune or tumor cells, or both.

The work from Bergman et al. is an important example of polyphenol pleiotropism when it comes to cytokines. A dose-dependent inhibition of proliferation of HT29 (human colon adenocarcinoma) and RKO (human colon carcinoma) cell lines was demonstrated after 24 h incubation with RES, with no effect on cytokine production. On the other hand, the incubation of PBMCs with RES resulted in reduced release of IL-6 and IL-10, a slight increase in TNF-α and no influence on IL-1β, IFN-γ and IL-1rα. The combination of PBMCs, RES and cancer cells or their conditioned media (CM) had variable effects but, as an example, the direct cell-to-cell contact of PBMCs and HT29, in presence of RES (60 µM), reduced the production of all the cytokines analyzed. Finally, the effects on cytokine production varied dose-dependently: for example, RKO co-cultured with PBMC in presence of different RES concentrations generally dropped the levels of IL-1β, IFN-γ and IL-10, but low RES doses stimulated TNF-α, while higher doses greatly reduced it [109]. Likewise, Falah et al. determined the efficacy of combinations of the anti-diabetic drug metformin (MET), with CUR on a set of different cell lines (EMT6/P, MCF7, T47D and Vero cell lines). Expectedly, both drugs inhibited cell growth and combination treatment had maximal effect, but again cytokine effects vary. The expression of vascular endothelial growth factor (VEGF) was significantly decreased after combined treatment (8 mM MET + 110 µM CUR) and after MET alone, while CUR alone only showed a trend for reduction. Similarly, in in vivo experiment on Balb/c mice injected with EMT6/P cells, the combination MET+CUR decreased tumor size but only trends for increase of IFN-γ and for significant decrease of IL-4 were detected, while IL-2 and IL-10 level did not change significantly [110]. More conclusive are, instead, the results from Lu et al. who, in the CUR-vaccine model (CUR-PEG + LCP-Trp2) of mice challenged with B16F10 melanoma cell line, evaluated cytokines levels in the tumor microenvironment and found a significant decrease of IL-6 and CCL2 and a dramatic increase in TNF-α and IFN-γ levels, helpful cytokines in the establishment of a cytotoxic T cell-mediated response [95]. Additionally, Orsolic et al. evaluated cytokine levels in the peritoneal fluid of mice injected with EAT cells and found a dose-dependent increase of Th1 cytokines IL-2, IL-12 and IFN-γ after progressive concentration of CA treatment. Concomitantly, Th2 cytokines IL-4 and IL-10 were found decreased [84]. Similarly, Lasso et al. detected an increased serum level of TNF-α and IL-6 and a decrease of IL-17 and IL-4 in in vivo melanoma mouse models treated with the P2Et prophylactic plus therapeutic protocol, while the breast cancer in vivo model did not show changes in cytokines production [91], strongly hinting at different immunoregulatory systems operating in mice (melanoma) and human (breast cancer) tumor microenvironment.

Intriguingly, the anti-inflammatory effect of polyphenols can also exert anti-tumoral activities. In this context, Mukherjee et al. demonstrated that, in prostate cancer cells (PC3, DU145, LnCap), EGCG acts by inhibiting tumor-supportive pro-inflammatory stimuli. Prostate cancer cells pre-treated with EGCG (40 µg/mL, 24 h) and then transfected with CpG-ODN (1 µM, 6 or 24 h) showed a generalized inhibition of mRNAs for inflammatory cytokines such as IL-6, IL-8, CXCL-1, IP-10, CCL-5, TGF-β1, therefore experiencing a modulation of pathways that drive tumor growth [111].

Results reporting analysis of cytokines expression are the trickiest to be discussed because is not undoubtedly defined the source of these molecules. Despite this not negligible detail, data collected here, tell of a contribution of secreted cytokines to a pro-inflammatory microenvironment, that awake and sustain immune cells in their tumor contrasting action. But, the need of a fine regulation of these molecules must be kept in mind because side effects can easily be generated.

### 4.6. Endothelial Cells

As proliferation and metastatic dissemination of cancer cells depend necessarily on oxygen and nutrient supply, chemopreventive compounds with the ability to target angiogenesis are of extreme interest, as they could complement immuno-modulatory activities and empower the final anti-cancer effect.

In this context, Yusuf et al. evaluated the mechanism of action of RES in dimethylbenzathracene (DMBA)-induced mouse models of skin cancer, with a particular focus on tumor vasculature investigated by analyzing the expression of endothelial cell marker CD31, angiogenesis factors VEGF and matrix metalloproteinase (MMP)-2 and MMP-9. Results showed effects depending on the mouse strain and the functionality of the immune receptor TLR4 but pointed to a decrease in all vascular markers in the mouse strain (C3HeN) competent for TLR4, linking RES effect on apoptosis and immune responses to its effect on neoangiogenesis [112]. Moreover, the findings by Guan et al. demonstrated RES-induced protection from therapy-provoked endothelial cells damage. High-dose IL-2 (HDIL-2) administration demonstrated to be a valuable instrument against metastatic melanoma but is associated with systemic toxicity in the vascular compartment. RES prevented the vascular leak syndrome (VLS) and reduced tumor growth and metastasis in animal model (B16F10 in C57BL/6 mice) treated with HDIL-2 + RES. The polyphenol acted reducing apoptosis of endothelial cells and promoting efficacy of therapy thanks to the induction of MDSCs, that suppressed lymphokine-activated killer cells (LAK), causative of vascular damage [113]. Additionally, the combination of CUR-PEG + LCP-Trp2 vaccine, as seen by Lu et al., had a great influence on tumor vessels, which were significantly reduced, and tumor-associated fibroblasts (TAFs), which were almost completely absent, confirming the compounds’ antiangiogenic effects [95]. Similar findings were reported by Orsolic et al., who observed significant inhibition of neovascularization, reduced micro vessel density and VEGF release in tumoral ascitic fluid of EAT-injected mice treated with CA [84]. In addition, a fraction rich in polyphenolic compound (termed A009) from olive mill wastewater (OMWW) was recently reported to significantly inhibit proliferation, morphogenesis and migration of human umbilical vein endothelial cells (HUVEC) in vitro, as well as angiogenesis in vivo [114]. The same fraction was also shown to inhibit the production of pro-angiogenic factors (VEGF, IL-8 CXCL8, CXCL12), in addition to proliferation, invasion and migration of human and murine colon cancer cells (HT-29, HCT-116, CT-26) or of prostate cancer cells (PC-3, DU-145, LNCaP), thus again linking anti-proliferative and ant-angiogenic effects together [115,116].

### 4.7. Immune Checkpoint

With the spreading of immune checkpoint inhibitor therapy in cancer, some polyphenols have been evaluated for their ability to affect the expression of immune checkpoint or to synergize with therapy. Data availability is too moderate to infer any conclusion, but it seems probable that yet again the effect of polyphenols is cell-, dose- and context-dependent.

Very recently, two publications evaluated the expression of PD-L1 after treatment with polyphenolic compounds. Xu et al. treated human melanoma cells (A375, A2058, RPMI-7951) with APG and CUR obtaining the inhibition of PD-L1 expression consecutive to IFN-γ-induced upregulation. In fact, IFN-γ supplementation greatly stimulated expression of PD-L1 by melanoma cell lines and both CUR and, mainly, APG lowered them, inhibiting STAT-1 phosphorylation. In a T cell-mediated melanoma cell killing assay, APG induced T cell to exert the higher cytotoxic capacity compared to control and to CUR. In a lactate dehydrogenase (LDH) release assay performed on co-culture (T cell/melanoma cells) this result was confirmed, and a further sign of T cell activation was the restored level of IL-2 in co-culture CM in the polyphenols treated groups. Melanoma xenograft (B16F10 on C57BL/6 mice), treated with CUR and APG, showed a significantly diminished growth of tumor implants harboring an increased immune cell infiltration (CD4^+^, CD8^+^), a reduced expression of PD-L1 on tumor cells and significantly lower percentage of Ki-67^+^ cells [117]. Lucas et al. instead treated with RES (50 µM) and piceatannol (Pic, 50 µM) a panel of breast (Cal51, BT549, BT474 and SKBR3) and colon cancer (HCT116, SW480, HT29 and SW620) cell lines. Positive responses to stimulation were observed in 2/4 breast and in 3/4 colon cancer cell lines individually treated with the polyphenols, while all the cells up-regulated PD-L1 when treated with a combination of the two compounds, through a mechanism involving IFN-γ—mediated activation of NF-kB. Notably, the combination of RES+Pic had heterogeneous effects, as it also induced DNA damage and downregulated p-38-MAPK and c-Myc levels in parallel with PD-L1 regulation. Furthermore, since PD-L1 expression can be regulated via histone deacetylases or histone acetyltransferases, inhibitors of these enzymes were tested and displayed lack of influence on PD-L1 expression. Surprisingly, however, if the inhibitor treatment was followed by polyphenol treatment, induction of PD-L1 expression was detected, thus hinting again at context-dependent effects of polyphenols on immune cells and activities [118]. Even more contrasting, Liao et al. evaluated the level of PD-L1 and p-STAT3^Y705^ expression in a model of oral squamous carcinoma treated with CUR, both in vitro and in vivo, and found the two molecules significantly downregulated by the treatment [100]. As cited, IFN-γ induces PD-L1 expression in tumor microenvironment, and Rawangkan et al. evaluated the modulation of immune checkpoint after EGCG and green tea extract (GTE, blend of cathechins) treatment. In vitro analysis on lung cancer A549 and H1299 cell lines showed a dose-dependent reduction of PD-L1 expression both at mRNA and protein level, because of inhibition of the JAK2/STAT1 signaling pathway. Moreover, EGCG can reduce PD-L1 expression also in Lu99 lung cancer cell line treated with EGF. Either the presence of EGF in the tumor microenvironment or mutations in its receptor (EGFR) are driver of immune checkpoint expression. On 4-(methylnitrosamino)-1-(3-pyridyl)-1-butanone (NNK)-induced lung tumors of A/J mice, GTE administration in drinking water reduced PD-L1 expression on tumor cells and tumor volume and, in co-cultures of B16F10 melanoma cells/tumor-specific T cells, EGCG reduced PD-L1 expression as well and maintained IL-2 transcription [119].

Finally, Shao et al. have evaluated the effect of anti-PD-L1 antibody in combination with the CUR natural analogue bisdemethoxycurcumin (BDMC) in an in vivo mouse model of bladder cancer, a type of neoplasia known to express PD-L1. Both a subcutaneous and a metastatic model, generated via intravenous injection of MB49 tumor cells, were taken into consideration and a reduction of tumor volume and increased mouse survival were reported, together with an increase in the frequency of CD8^+^ T cells in the spleens and in the tumor-draining lymph nodes of both models. Moreover, CD8^+^ T cells also displayed increased antitumoral activity, showing a reduction of PD-1 expression accompanied by higher levels of IFN-γ and granzyme B in the group treated with BDMC [120].

## 5. Conclusions

With more than 8000 known polyphenols found in foods such as tea, wine, chocolate, fruits and vegetables [121], the potential for nutraceutical use of these compounds is enormous and the interest in their use as chemopreventive and chemosupportive drugs is ever growing. Polyphenols are generally regarded as anti-inflammatory, antioxidant molecules with a several beneficial effects on metabolism and a great applicability in cancer, where they could both fight the oncogenic drive of free radicals and suppress chronic local inflammation that maintains and sustains tumor growth.

However, accumulating evidence points to a more complex scenario: polyphenol effects depend, in fact, on the state of target cells (e.g., resting versus activated), on their ontogeny and pathological condition (e.g., normal versus cancer cells or macrophages versus TAMs) and on the dosage, exposure times and pharmacokinetics. Hence, a more solid understanding of polyphenol activities at the system biology level is needed to develop novel chemotherapeutic combinations to which these compounds could provide significant value. A particular area of interest in this context is immunotherapy, as polyphenols could beneficially boost anti-tumoral immunity while abrogating or delaying the rise of tumor-supporting leukocytes. Considering that immunotherapy is only now weighting its enormous potential in “mainstream” oncology, it is unsurprising to notice that studies on the effects of polyphenols in immunotherapy are just at their dawning.

Recapitulating, polyphenols show promising activities as immunomodulatory molecules and this suggests, in turn, that more studies should be devoted to systematically analyze the usability of polyphenols in immunotherapeutic protocols. On the other hand, some indications exist that polyphenols could also exert detrimental activities, calling for caution in considering them as “natural allies” in immunotherapy.

Future studies, focused on exact immunotherapeutic protocols and well-defined cell and animal models, will probably solve the current controversies and help us exploring new ways to fight cancer.

## Figures and Tables

**Table 1:** In vitro and in vivo effect of polyphenols on immune cells.

	Cell Type	Treatment	In Vitro Model	In Vivo Model	Effect on Immune System	Ref.
MOUSE	SPLENOCYTES	CHR	♂ Wistar rat, LPS o lectin-stimulated, 3–25 µM, 48 h		↓ proliferation (LPS) ↑ proliferation (lectin)	[78]
		CUR	♀ Balb/c, + ConA 1 µg/mL or LPS 5 µg/mL + CUR 1–20 µM, 72 h		↓ T cell proliferation (ConA) ↓ IL-4, IFN-γ secretion ↓ B cell proliferation (LPS) ↓ IgG1, IgG2 production ↔ viability	[88]
		HSP	♂ Wistar rat, LPS or lectin-stimulated splenocytes, 3–25 µM, 48 h		↓ proliferation (LPS) ↓ proliferation (lectin)	[78]
		JSE	♂ C57BL/6, 1–200 µg/mL, 48 h		↑ proliferation	[85]
		RES	♀ Balb/c, + ConA 1 µg/mL or LPS 5 µg/mL + RES 1–20 µM, 72 h		↓ T cell proliferation (ConA) ↓ IL-4, IFN-γ secretion ↓ B cell proliferation (LPS) ↓ IgG1, IgG2 production ↔ viability	[88]
			IL2 + ConA stimulation	♀ A/J bearing neuroblastoma (NXS2) s.c., 20 mg p.t./every 3 days	↔ circulating leukocyte population ↑ tumor infiltrating leukocytes (CD45^+^) ↓ splenocytes proliferation ↓ ADCC	[76]
			♂ C3H (H-2k) splenocytes, IL-2 or ConA-stimulated + RES 6, 25–50 µM	♂ C3H (H-2k) RES p.o. 2 mg/day, 5 days/week, 4 weeks	↑ proliferation (RES 6.25–12.5 µM) ↓ proliferation (RES 25–50 µM) ↔ body weight ↔ peripheral blood cell count ↔ IFN-γ secretion (ConA-stimulated splenocytes)	[79]
HUMAN	PERIPHERAL BLOOD MONONUCLEAR CELLS	RES	PBMC healthy donor, 0–60 µM		↓ IL-6, IL-10; ↑ TNF-α; ↔ IFN-γ, IL-1ra, IL-1b	[109]
		HT	PBMC healthy donor, pre-treated HT 0.25–1 µM, 30′ + treated Oxysterols mixture 20 µM, 24 h		↓ IL-1b, MIF, RANTES ↓ intracellular ROS production ↓ p-JNK1/2	[75]
		TYR	PBMC healthy donor, pre-treated TYR 0.25–1 µM, 30′ + treated Oxysterols mixture 20 µM, 24 h		↓ IL-1b, MIF, RANTES ↓ intracellular ROS production ↓p-JNK1/2	[75]
		HVA	PBMC healthy donor, pre-treated HVA 0.25–1 µM, 30′ + treated Oxysterols mixture 20 µM, 24 h		↓ IL-1b, MIF, RANTES ↓ intracellular ROS production ↓p-JNK1/2 (1 µM)	[75]
		RES	PBMC healthy donor, PHA stimulated, 1–50 µM		↓ PBMC proliferation ↓ ADCC	[76]
			PBMC healthy donors, HT29, 0–60 µM		↓ IL-6, IL-10, TNF-α, IFN-γ, IL-1ra, IL-1b	[109]
			PBMC healthy donors, RKO, 0–60 µM		↓ IL-1b, IFN-γ, IL-10 ↔ IL-6 and IL-1ra ↑ TNF-α	[109]
		Linalool	Lymphocytes healthy donor, 227 µM, 24 h		↑ CD40-ligand, CD40, IFN-γ, IL-12 p40, IL-13, IL-17F, IL-1β, IL-2, IL-21, IL-21R, IL-23p19, IL-4, IL-6Sr, TNF-α	[77]
		EGCG	Breast cancer patients, PBMC stimulated with PHA, anti-CD3, or Her2/neu and p53 antigen peptides, EGCG 0.125–50 µg/mL		↓ PBMC proliferation > 10 µg/mL ↓ IFN-γ production > 10 µg/mL	[80]
MOUSE	MACROPHAGES	CA		♂ Swiss albino bearing Ehrlich ascites tumor (EAT) cells	↑ macrophages count ↑ macrophages cytotoxicity ↓ ARG1; ↔ NO ↔ neutrophils, lymphocytes count	[84]
		CHR	♂ Wistar rat, LPS o lectin-stimulated splenocytes, 3–25 µM, 48 h		↓ macrophage lysosomal enzyme activity ↓ NO production	[78]
		CUR	♀ Balb/c, peritoneal macrophages, LPS stimulated + 1–20 µM, 48 h		↓ IL-1, IL-6, TNF-α ↑ IL-10 ↓ CD80, CD86 expression ↔ CD40 expression	[88]
		EGCG		♂ Balb/c bearing leukemia cells (WEHI-3), 5–40 mg/kg p.o.	↑ phagocytosis	[81]
				Balb/c bearing mammary cancer cells (4T1) s.c., EGCG i.p. 10 mg/kg, day 7-day 9-day 11	↓ infiltration TAM and M2 ↓ CSF-1, CCL-2 ↓ IL-6, TGF-β ↑ TNF-α	[82]
		HSP	♂ Wistar rat, LPS o lectin-stimulated splenocytes, HSP 3–25 µM, 48 h		↑ macrophage lysosomal enzyme activity ↔ NO production	[78]
		P2Et	Melanoma cells (B16F10), 72.7 µg/mL, 36 h	C57BL/6 bearing melanoma cells (B16F10) s.c., 75 mg/mL	↑ CD45^+^CD220^−^CD11c^+^ ↑ CD86, CD40, MHCII, CD70 ↑ BMDCs phagocytosis	[92]
				♀ C57BL/6 Healthy, 75 mg/kg i.p., twice/week/3weeks	↑ DCs, ↑ MDSC-LC	[91]
				♀ Balb/c Healthy, 75 mg/kg i.p., twice/week/5 weeks	↑ DCs ↑ MDSC-LC	[91]
		RES	♂ C57BL/6, EG7 cells, BMDC pre-treated 1 h, 20–50 µM + 18 h IFN-γ 100 U/mL		↓ IDO expression ↓ IRF-1 expression ↓ STAT1, PKCδ	[89]
			♀ Balb/c, peritoneal macrophages, LPS stimulated + 1–20 µM, 48 h		↓ IL-1, IL-6, TNF-α ↑ IL-10 ↓ CD80, CD86 expression ↔ CD40 expression	[88]
		Rutin		♂ Balb/c bearing leukemia cells (WEHI-3), macrophages from PBMC or peritoneum, 6–12 mg/kg, p.o., 3 weeks	↑ phagocytosis	[83]
		TrLp		♂ C57BL/6 bearing Glioblastoma cells (GL261) i.c. implanted, TrLp 1,28 mM, i.p., every 24 h, 5 days	Switch from M2-like TAM ARG1^high^iNOS^low^ to M1-like TAM phenotype ARG1^low^iNOS^high^ ↑ NO production	[87]
				C57BL/6 bearing Lung cancer cell (HPV^+^ TC-1), 64 µM, i.t. infusion every 24 h, 5 days	‘tumor-core’: E6^+^ tumor cells, ‘tumor-periphery’: Iba1^+^ TAM Switch from ARG1^high^iNOS^low^IL-12^low^IL-10^high^ M2 TAM to ARG1^low^iNOS^high^IL-12^high^IL-10^low^ M1 TAM ↑ NO ↓ p-STAT3 ↑ p-STAT1, p-NF-Kb	[86]
HUMAN	MACROPHAGES	JSE		JSE, 1–200 µg/mL, 48 h	↑ phagocytosis, ↑ NO, ↑ H_2_O_2_	[85]
		TrLp		HNSCC cells (UMSCC47); Nu/nu mice i.t. TrLp thrice/week/5weeks	Switch from ARG1^high^iNOS^low^ Iba1^+^ M2 Macrophages to Iba1^+^ TAM ARG1^low^iNOS^high^ M1 ↓ p-STAT3 ↑ p-STAT1, p-NF-kB ↑ NO	[86]
		Nano-CUR		0–50 µM, 48 h	↔ CD80, CCR7 ↑ CD86 ↔ TNF-α, IL-6, IL-12	[94]
MOUSE	T LYMPHOCYTES	CHR or HSP	♂ Wistar rat, LPS o lectin-stimulated splenocytes, CHR or HSP 3–25 µM, 48 h		↑ CTL activity against B16F10	[78]
		CUR	♀ Balb/c splenocytes, + ConA 1 µg/mL + CUR 1–20 µM		↓ CD28 expression on CD4 ↑ CTLA-4 expression on CD4 ↓ proliferation T cells ↓ IFN-γ, IL-4 secretion	[88]
				♀ Balb/c bearing mammary cancer cells (TUBO) s.c., CUR p.o. 2 mg/50 µL oil, 3 days/week, ± CQ 2 mg/50 µL water, 5 days/week	↑ CD8, ↓ Foxp3 Treg cells ↓ CD8, ↑Foxp3 Treg cells, (CUR+CQ)	[97]
				♀ C57BL/6 bearing oral carcinoma 4NQO-induced in drinking water for 16 weeks, CUR 4 weeks	↑ CD8 in tumor microenvironment	[100]
			♀ C57BL/6 bearing Lewis lung carcinoma (LLC), splenic lymphocytes isolation, activation and treatment with CUR 1.5 µg/mL, 48 h		↑ frequency and number of T cells (CUR < 1.5 µg/mL) ↔ frequency and number of B cells, DCs, NK ↓ frequency and number of T cells (CUR > 2 µg/mL)	[101]
				♀ C57BL/6 bearing Lewis lung carcinoma cells (LLC), CUR 0–100 mg/kg/day/mouse, 10 days, i.p.	↑ CD8 cytotoxicity and proliferation (CUR < 1.5 µg/mL) ↑IFN-γ	[101]
			♂ C57BL/6 BMDC LPS-matured, treated CUR 0–25 µM, 45′		↓ BMDCs maturation ↓ CD80, CD86, MHCII ↓ IL-1, IL-6, TNF-α ↓ T cell activation ↓ IFN-γ production	[102]
		EGCG		♂ Balb/c bearing leukemia (WEHI-3) cells, 5–40 mg/kg p.o.	↑ CD3^+^	[81]
		P2Et		Vaccination with melanoma cells (B16F10) pre-treated with 101.6 µg/mL P2Et, 48 h	↑ CD8^+^CD44^+^, CD8^+^CD44^+^CD62L^+^ ↑ CD8^+^ IFN-γ^+^	[92]
				♀ C57BL/6 bearing melanoma cells (B16F10), 75 mg/kg, i.p. twice/week/10 days + s.c. twice/week/21 days	↓ CD3^+^, CD4^+^, CD8^+^ (LN) ↓ CD44^+^ (LN)	[91]
				♀ C57BL/6 Healthy, 75 mg/kg i.p., twice/week/3 weeks	↑ CD4^+^, CD8^+^	[91]
				♀ Balb/c bearing mouse mammary cancer cells (4T1) cells, 75 mg/kg, i.p. twice/week/10 days + s.c. twice/week/32 days	↓ CD3^+^, CD4^+^, CD8^+^ (LN) ↔ CD44^+^ (LN) ↓ CD4^+^ T_EM_↑ CD8^+^ T_N_↓ CD8^+^ T_EM_	[91]
				♀ Balb/c Healthy,75 mg/kg i.p., twice/week/5 weeks	↑ CD4^+^, CD8^+^	[91]
		RES		♀ Balb/c bearing renal adenocarcinoma cells (RENCA), 1–5 mg/kg, i.p.	↑ CD8 density; ↑CD69^+^ ↑ CD8 Perforin, Granzyme B, FasL	[93]
			♀ C57BL/6 spleen of T cell lymphoma (EG7)-bearing mice, 25–75 µM RES, 24 h	♀ C57BL/6, i.p. 4 mg/kg RES	↑ CD8^+^IFN-γ^+^	[103]
			♂ C57BL/6 OT-1; CD8 co-cultured with DC pulsed with OVA + 18 h IFN-γ 100 U/mL		↑ CD8 proliferation ↑ CTL activity	[89]
			♀ Balb/c splenocytes, ConA, RES 1–20 µM		↓ CD28 expression on CD4^+^ ↔ CTLA-4 expression on CD4^+^ ↓ proliferation T cells ↓ IFN-γ, IL-4 secretion	[88]
		Rutin		♂ Balb/c bearing leukemia cells (WEHI-3), macrophages from PBMC or peritoneum, 6–12 mg/kg, p.o., 3 weeks	↑ CD3, CD19 ↓ CD11b, Mac3	[83]
		TrLp		C57BL/6 bearing Lung cancer cell (HPV^+^ TC-1), TrLp 64µM, i.t. infusion/every 24 h/5 days	↑ CD8^+^ CTL	[86]
HUMAN	T LYMPHOCYTES	Nano-CUR		0–50 µM, 48 h	↔ phenotype resting T cells ↔ cytokine production in resting T cells ↓ TNF-α, IL-6, IL-8, IL-10, IL-1b in activated T cells	[94]
			Oral carcinoma (OE19), 50 µM, 48 h		↑ CTLs lysis ↔ TNF-α, IL-8, IFN-γ, IL-2	[94]
			Oral carcinoma (OE33), 50 µM, 48 h		↑ CTLs lysis ↑ IFN-γ ↓ IL-8 ↔ TNF-α, IL-2	[94]
MOUSE	T REGULATORY CELLS AND MIELOID DERIVED SUPPRESOR CELLS	CUR	♀ Balb/c splenocytes, + ConA 1 µg/mL + CUR 1–20 µM		↔ frequency CD4^+^ CD25^+^ Treg cells	[88]
				♀ C57BL/6 bearing oral carcinoma 4NQO-induced in drinking water for 16 weeks, CUR 4 weeks	↓ Treg cells (CD4^+^ CD25^+^ Foxp3^+^) ↓ MDSCs (CD11b^+^ GR1^+^) ↑ CD8^+^ in tumor microenvironment ↓ PD-L1, p-STAT3	[100]
				♀ C57BL/6 bearing melanoma cells (B16F10) s.c., treated with CUR-PEG and LCP Trp2-based vaccine	↓ MDSCs ↓ Treg cells ↑ CD8+	[95]
				♀ C57BL/6 bearing Lewis lung carcinoma cells (LLC), 50 mg/kg p.o. daily	↓ MDSCs ↓ ARG1, iNOS, ROS ↑ F4/80, MHCII, CD80, CD11c ↑ CD8^+^, CD4^+^ ↓ IL-6 in tumor microenvironment	[105]
		P2Et		♀ C57BL/6 bearing melanoma cells (B16F10) cells, 75 mg/kg, i.p. twice/week/10 days + s.c. twice/week/21 days	↑ MDSC-LC cells	[91]
				♀ C57BL/6 Healthy, 75 mg/kg i.p., twice/week/3 weeks	↑ CTLA-4^+^, Foxp3^+^ Treg cells	[91]
				♀ Balb/c bearing mammary cancer cells (4T1) cells, 75 mg/kg, i.p. twice/week/10 days + s.c. twice/week/32 days	↑ MDSC-LC cells	[91]
				♀ Balb/c Healthy,75 mg/kg i.p., twice/week/5 weeks	↑ CTLA-4^+^, Foxp3^+^ Treg cells	[91]
		RES		♀ Balb/c bearing renal carcinoma cells (RENCA) cells, i.p. 1–5 mg/kg	↔ frequency	[93]
				♀ Balb/c bearing renal carcinoma cells (RENCA) cells, i.p. 1–5 mg/kg	↓ frequency	[93]
			♀ Balb/c splenocytes, + ConA 1 µg/mL + RES 1–20 µM		↔ frequency CD4^+^ CD25^+^ Treg cells	[88]
			♀ C57BL/6 spleen of T cell lymphoma (EG7)-bearing mice, 25–75 µM RES, 24 h	♀ C57BL/6 bearing T cell lymphoma (EG7), i.p. 4 mg/kg	↓ CD4^+^ CD25^+^ FoxP3^+^ ↓ CD4^+^CD25^+^/CD4^+^ ratio ↓ TGF-b ↑ CD8+IFN-γ^+^	[103]
			♀ Balb/c spleen of colon cancer (CT-26)-bearing mice, 25–75 µM, 24 h		↓ CD4^+^ CD25^+^ FoxP3^+^ ↓ CD4^+^CD25^+^/CD4^+^ ratio	[103]
HUMAN	T REGULATORY CELLS	CUR		PBMC from Lung cancer patients treated 3 g/day, 2 weeks	convert (CD4^+^CD25^+^Foxp3^+^) Treg cells into IFN-γ^+^ Th1 cells	[106]
				PBMC from advanced colon carcinoma patients treated 3 g/day, 2 weeks	↓ Treg cells (CD4^+^CD25^+^Foxp3^+^) ↑ CD4^+^CD25^+^Foxp3^−^, IFN-γ^+^	[106]
		EGCG, GTE		CLL patients (Rai stage 0), 6 months, 4602 mg of green tea leaves, 189 mg EGCG, 97,5 mg caffeine	↓ circulating lymphocytes and Treg cells ↓ IL-10 and TGF-β	[108]
		RES		Healthy subjects, 1 g/day, 4 weeks	↑ circulating Treg cells (CD3^+^CD4^+^CD25^+^CD127^dim/neg^) ↑ γδ^+^ NKG2D^+^ T cell ↑ CD3^−^CD56^+^ NKG2D^+^ NK ↔ CD8, CD4, CD19 ↓ TNF-α, MCP-1	[104]
MOUSE	CYTOKINES	CA		♂ Swiss albino bearing Ehrlich ascites tumor (EAT) cells	↑ IL-2, IL-12, IFN-γ ↓ IL-4 and IL-10	[84]
		CUR		♀ Balb/c bearing mammary cancer cells (EMT6/P) s.c., Met 100 µL 80 mg/kg i.p. + CUR 100 µL 50 mg/kg p.o.	↑ IFN-γ, IL-4 ↔ IL-2, IL-10	[110]
				♀ C57BL/6 bearing melanoma cells (B16F10) s.c., treated with CUR-PEG and LCP Trp2-based vaccine	↓ IL-6, CCL2 in tumor microenvironment ↑ TNF-α, IFN-γ in tumor microenvironment ↑ CTL response	[95]
		P2Et		♀ C57BL/6 bearing melanoma cells (B16F10) s.c., 75 mg/kg, i.p. twice/week/10 days + s.c. twice/week/21 days	↑ TNF-α, IL-6 ↓ IL-17, IL-4	[91]
				♀ Balb/c bearing mammary cancer cells (4T1) s.c., 75 mg/kg, i.p. twice/week/10 days + s.c. twice/week/32 days	↑ TNF-α, IL-6	[91]
				♀ C57BL/6 Healthy, 75 mg/kg i.p., twice/week/3 weeks	↑ IL-10, IL-17, IFN-γ, IL-6, IL-4, IL-2	[91]
				♀ Balb/c Healthy,75 mg/kg i.p., twice/week/5 weeks	↑ IFN-γ, IL-6	[91]
HUMAN	CYTOKINES	RES		♂ healthy subject, 5 g, orally administered	↑ Kynurenin/tryptophan ratio as measure of IDO activity	[90]
		EGCG	Prostate cancer cells (PC3, DU145, LnCap), pre-treated EGCG 40 µg/mL, 24 h and then transfected with CpG-ODN 1 µM, 6 or 24 h		↓ IL-6, IL-8, CXCL-1, IP-10, CCL-5, TGF-β1	[111]
MOUSE	ENDOTHELIAL CELLS	CA		♂ Swiss albino bearing Ehrlich ascites tumor (EAT) cells	↓ neovascularization ↓ reduced microvessel density ↓ VEGF release in ascite	[84]
		CUR		♀ C57BL/6 bearing melanoma cells (B16F10) s.c., treated with CUR-PEG and LCP Trp2-based vaccine	↓ tumor vessels ↓ TAF	[95]
		P2Et	Melanoma cells (B16F10), P2Et 1.9–250 µg/mL		↑ ICD, ↑autophagy	[92]
		RES		♀ C3/HeN or C3/HeJ (TLR4 mutated) bearing skin cancer DMBA-induced, 10 µmol/mouse, topically applied 1h before DMBA	↑ IFN-γ^+^ (skin lysates) ↑ IL-12 (skin lysates) ↓ MMP-2 MMP-9 ↓ VEGF (skin lysates) ↓ CD31 in tumor	[112]
				♀ C57BL/6 bearing melanoma cells (B16F10) s.c. or i.v., HDIL-2 75.000 U, 3 times/day/3 days + RES 100 mg/kg p.o., 5 days	↓ vascular leak syndrome	[113]
				♀ Balb/c bearing renal carcinoma cells (RENCA) cells, i.p. 1–5 mg/kg	↑ IFN-γ, Fas ↓ IL-6, IL-10	[93]
HUMAN	ENDOTHELIAL CELLS	A009	Human umbilical vein endothelial cells (HUVEC)		↓ proliferation ↓ angiogenesis ↓ migration	[114]
			Colon cancer cells (HT-29, HCT-116);Prostate cancer cells (PC-3, DU-145, LNCaP)		↓ VEGF, IL-8 CXCL8, CXCL12 ↓ proliferation ↓ angiogenesis ↓ migration	[115,116]
MOUSE	IMMUNE CHECKPOINT	APG or CUR		♀ C57BL/6 bearing melanoma cells (B16F10) s.c., CUR 50 mg/kg or APG 150 mg/kg, p.o., 12 days	↓ PD-L1 ↑ CD4^+^ CD8^+^ in tumor microenvironment	[117]
		EGCG or GTE	Melanoma cells (B16F10) co-culture with tumor specific T cells, EGCG 30 µM	♀ A/J mice bearing lung cancer NNK-induced; GTE 0.3% in drinking water	↓ PD-L1	[119]
		BDMC		♀ C57BL/6 bearing bladder cancer cells (MB49) s.c./i.v. treated with Anti-PD-L1 Ab + BDMC 3 mg/kg, 4/2 weeks	↑ CD8^+^ (SPL and LN) ↑ IFN-γ, granzyme B ↓ PD-1 ↑ survival	[120]
HUMAN	IMMUNE CHECKPOINT	APG or CUR	Melanoma cell lines (A375, A2058, RPMI-7951) treated APG 5–60 µM or CUR 5–30 µM, 24 h; Jurkat cell-mediated A375 killing assays		↓ PD-L1 ↓ p-STAT1 ↑ CD8 citotoxicity ↑ IL-2	[117]
		RES or Pic	Breast cancer cells (Cal51, BT549, BT474) and Colon cancer cells (SKBR3, HCT116, SW480, HT29 and SW620)RES 50 µM, Pic 50 µM		↑ PD-L1 ↑ DNA damage	[118]
		EGCG or GTE	Lung cancer cells (A549, H1299, Lu99), 50–100 µg/mL		↓ PD-L1	[119]

Abbreviations: i.c., intracranial; s.c., subcutaneous; i.p., intraperitoneal; i.t., intratumoral; p.t., peritumoral; p.o. per os; i.v. intra venous; LN, lymph node; SPL, spleen; NO, nitric oxide; LPS, Lipopolysaccharide; ROS, reactive oxygen species; ConA, Concanavalin A; Ab, antibody; CTL, cytotoxic T lymphocyte; DMBA, Dimethylbenzathracene; Met, Metformin; 4NQO, 4-nitroquinoline-oxide-induced; CQ, Chloroquine.
